# Sulfiredoxin-1 protects against simulated ischaemia/reperfusion injury in cardiomyocyte by inhibiting PI3K/AKT-regulated mitochondrial apoptotic pathways

**DOI:** 10.1042/BSR20160076

**Published:** 2016-04-27

**Authors:** Jiankai Zhang, Zhangyou He, Jinhua Guo, Zhe Li, Xiaohong Wang, Chun Yang, Xiaojun Cui

**Affiliations:** * Department of Human Anatomy, Institute of Stem Cell and Regenerative Medicine, Guangdong Medical University, Zhanjiang 524023, Guangdong, China; † Internal Medicine, Shenzhen Guangming New District Central Hospital, Shenzhen 518107, Guangdong, China

**Keywords:** cardiomyocyte, ischaemia/reperfusion injury, mitochondrial apoptotic pathways, PI3K/AKT, sulfiredoxin-1

## Abstract

Reactive oxygen species (ROS)-triggered cardiac cell injury is recognized as the major contributor for the pathogenesis progression of ischaemic cardiovascular diseases. Sulfiredoxin-1 (Srx-1) is an endogenous antioxidant and exerts the crucial neuroprotective effects in cerebral ischaemia. However, its function and the underlying mechanism in ischaemic heart diseases remain poorly defined. Here, a dramatical decrease in Srx-1 was validated in H9c2 cardiomyocytes upon simulated ischaemia–reperfusion (SI/R) injury. Moreover, Srx-1 protected H9c2 cells from SI/R-injured injury as the evidences that Srx-1 up-regulation attenuated the inhibitory effects on cell viability, lactate dehydrogenase (LDH) and cell apoptosis upon SI/R treatment. Knockdown of Srx-1 accelerated cell injury upon SI/R. Mechanism assay corroborated that SI/R treatment noticeably aggravated the loss of mitochondrial membrane potential (Δ*ψ*_m_), which was remarkably abated in Ad-Srx-1 groups. Importantly, Srx-1 elevation substantially reduced cytochrome *c* release, the activity of caspase-9 and caspase-3, accompany with the subsequent decrease in the cleavage of poly (ADP ribose) polymerase (PARP). Concomitantly, overexpression of Srx-1 also decreased the expression of pro-apoptosis protein Bax and increased anti-apoptotic Bcl-2 expression. Further analysis substantiated that Srx-1 treatment remarkably induced the activation of PI3K/AKT signalling. Preconditioning with LY294002 dramatically decreased Srx-1-enhanced cell resistance to SI/R injury. Importantly, LY294002 mitigated the inhibitory effects of Srx-1 on Δ*ψ*_m_ loss, cytochrome *c* release, caspase-9/3 activity, and the expression of Bcl-2 family. Together, these results suggested that Srx-1 might protect cardiomyocyte injury upon SI/R by suppressing PI3K/AKT-mediated mitochondria dependent apoptosis, revealing a promising therapeutic agent against ischaemic cardiovascular diseases.

## Abstract (precis)

The present study confirmed that Srx-1 overexpression could protect cardiomyocyte from SI/R-induced injury by suppressing PI3K/AKT-regulated mitochondria dependent apoptosis. Therefore, the present study will support a promising therapeutic avenue for the treatment of ischaemic cardiovascular diseases.

## INTRODUCTION

Ischaemic cardiovascular diseases are the leading cause of the life-threatening with the high morbidity and mortality in the developing world [1]. They rank second for mortality in Korea and result in approximate 4 million deaths in Europe, as well as 1 million new patients in China each year [2,3]. Recently, they exhibit the increasing influence on younger population in contrast with traditional occurrence in elderly people [2]. Restoring blood supply is known as the critical step for the current treatments of ischaemic cardiovascular diseases [4–6]. However, this reperfusion can lead to further myocardial ischaemia–reperfusion (I/R) injury, which is a major contributor for myocardial stunning, malignant arrhythmia and cardiac failure [7,8]. Furthermore, I/R myocardial injury can trigger the left ventricular remodelling and electrophysiological reconstitution, resulting in heart failure and subsequent sudden myocardial infarction [9]. Though the large of significant therapeutic advances, I/R myocardial injury remains a major unsolved public health problem.

Abundant researches have shown that the high production of reactive oxygen species (ROS) cause oxidative stress, which may contribute to the pathogenesis progression of cardiovascular diseases such as heart failure and myocardial I/R injury [10–12]. Oxidative stress damage often causes the balance disorders between the endogenous antioxidant defense and clean system, leading to cardiac cell injury. Therefore, preventing oxidative stress injury maybe a desirable endpoint for the therapeutic strategy to myocardial injury. Sulfiredoxin-1 (Srx-1) belongs to the sulfiredoxin family of antioxidants and plays the important role in various physiological processes, including cell apoptosis, invasion and redox balance [13–15]. As a central endogenous antioxidant, Srx-1 was initially identified in yeast [16]. Over the past few years, increasing evidences have confirmed the critical role of Srx-1 in regulating oxidative stress-triggered cell damage [13,17]. It has been corroborated that the induction expression of Srx-1 contributes to neuroprotective ischaemic preconditioning under the conditions of oxidative insults [14]. When Srx-1 expression was transiently knocked down in PC12 cells, cellular damage increased and cell viability decreased, indicating a potential target for neuroprotective intervention in response to oxidative stress [17]. However, its function and the related regulatory mechanisms involved in myocardic I/R injury remain poorly elucidated.

Accordingly, the objective of the present study was to explore the effects of Srx-1 overexpression or silence on cardiomyocyte viability and apoptosis under simulate ischaemia–reperfusion (SI/R) injury. Moreover, the underlying mechanism was also investigated.

## MATERIALS AND METHODS

### Chemicals and antibodies

The PI3 kinase inhibitor LY294002 was obtained from Cayman. The primary antibodies against poly (ADP ribose) polymerase (PARP) and caspase-3 were purchased from Cell Signaling Technology. Rabbit polyclonal anti-cytochrome *c* and anti-Srx-1 antibodies were from Abcam and Bioss, separately. The antibodies against caspase-9, Bax and Bcl-2 were acquired from Santa Cruz Biotechnology. The antibodies against p-Akt (Ser-473), p-Akt (Thr-308) and AKT were from Cell Signaling Technology.

### Cell culture

Rat embryonic cardiomyocyte cell line H9c2 was purchased from A.T.C.C. Cells were maintained in DMEM medium supplemented with 10% fetal bovine serum (FBS), 100 U/ml penicillin G and 100 μg/ml streptomycin. All cells were incubated in a humidified atmosphere with 5% CO_2_ at 37°C.

### Adenovirus construction

The full length of rat Srx-1 cDNA fragments was amplified and then was sub-cloned into the adenoviral shuttle plasmid pAdTrack-CMV (Agilent) containing green fluorescent protein (GFP). Then, the recombinant pAdTrack-CMV-Srx-1-GFP was homologously recombinated with the adenoviral backbone vector pAdEasy-1 in *Escherichia coli* strain BJ5183. Insert orientation was assessed by DNA sequencing (Sangon). The obtained recombinant plasmids were transfected in HEK293T cells (A.T.C.C.) to generate the recombinant Ad-Srx-1 adenovirus using Lipofectamine 2000 (Invitrogen). After large-scale virus propagation in 293T cells, virus were purified by banding twice on CsCl gradients. The virus titers were determined using p24 ELISA kit (Cell Biolabs).

### Srx-1 silencing by RNA interference

To knockdown Srx-1 expression in H9c2 cells, the small interference RNAs (siRNAs) targeting Srx-1 and scramble siRNA were designated as previously reported [17]. The scramble siRNA (siRNA-con) was used as a negative control. siRNAs targeting Srx-1 were 5′-GCATCGACACTGTGCACAA-3′. Both the fragments of above siRNA were synthesized by Shanghai Sangon. For siRNA transfection experiments, cells were seeded in 24-well plates. Then, 2 μg/ml of siRNAs were transfected into cells with the help of RNAi Max (Invitrogen) according to manufacturer's directions. Following 24 h incubation, the knockdown efficiency was evaluated by qRT-PCR and western blotting.

### Simulated ischaemia–reperfusion treatment

H9c2 cells were incubated in the presence of 2 nmol/l Ad-Srx-1 adenovirus at 37°C, or Ad-GFP. Approximately 48 h later, cells were subjected to SI/R. Specifically, the medium were replaced with serum- and glucose-deficient DMEM. Then, cells were placed into a chamber mimicking hypoxia containing 1% O_2_, 94% N_2_ and 5% CO_2_. After 10 h incubation, re-oxygenation was performed in DMEM medium including 10% FBS for 3 h at 37°C.

### RNA extraction and real-time quantitative RT-PCR (qRT-PCR)

To quantify mRNA expression, total RNA from different specimens were obtained using RNAiso Plus (Takara), followed by the reverse transcription into the first strand cDNA with High-Capacity cDNA Reverse Transcription Kits (Applied Biosystems). The obtained cDNA was then subjected to qRT-PCR analysis using SYBR Premix Ex TaqTM II Kit (Takara) in accordance with the manufacturer's standard protocols. The specific primers for rat Srx-1 were used as previously reported [13] and obtained from Sangon. β-Actin was used as a control to normalize gene expression, and results were calculated using 2^−ΔΔCt^.

### Western blotting

Total protein was extracted from cells using RIPA lysis buffer (Beyotime), and protein concentrations were measured by BCA protein assay kit (Beyotime). Then, 200 μg of protein per lane was separately electrophoresed by SDS/12% PAGE, followed by the electroblotting on to a PVDF membrane (Schleicher & Schuell). After incubation with 5% nonfat dry milk in PBS to block the non-specific bind, the membranes were immunoblotted with the primary antibodies against Srx-1, cytochrome *c*, caspase-3, caspase-9, PARP, Bax, Bcl-2, p-Akt (Ser-473), p-Akt (Thr-308) and AKT at 4°C overnight. After washing with TBST, membranes were incubated with corresponding secondary antibodies conjugated to horseradish peroxidase. The binding signals were visualized by enhanced chemiluminescence reagent (ECL; Santa Cruz Biotechnology) and band intensities were quantified using Quantity One (Bio-Rad Laboratories).

### Cell viability

Cell viability was assessed by MTT colorimetric analysis. Briefly, cells were seeded into 96-well plate at 2.0×10^5^ cells/well. Cells were treated with the PI3K/Akt inhibitor LY294002 prior to transfection with Ad-Srx-1 under SI/R stimulation. Then, 100 μl of 0.5 mg/ml MTT solution (Sigma) was added. After incubation for 3 h, 100 μl of DMSO was added to dissolve the formazan precipitate. The collected samples were subjected to ELISA reader to measure the absorbance *D* values at 570 nm. Relative cell viability was expressed as percentage of the control group.

### Annexin V/propidium iodide (PI) staining

Cells from the above different groups were collected and washed with PBS three times. After centrifugation, cells were re-suspended with 500 μl binding buffer, followed by the incubation with 10 μl Annexin V-FITC and 5 μl PI (Beyotime). The above reaction was performed at room temperature in the dark. Approximately 15 min later, cells were subjected to FACScan flow cytometer (BD Biosciences) for quantitative apoptosis assay.

### Cytochrome *c* detection

Cells from various experimental groups were collected and washed with ice-cold PBS. Then, cells were homogenized in RIPA buffer (Sigma) including 1% protease inhibitor cocktail. After 30 min on ice, the specimens were centrifuged at 12000 × ***g*** for 20 min at 4°C. The obtained protein concentrations were detected by a Bio-Rad DC protein assay kit (Bio-Rad Laboratories). The cytochrome *c* release into cytosol was then evaluated by western blotting.

### Caspase enzyme activity assay

Caspase-3 and caspase-9 activities were detected by Caspase-3 Fluorescent Assay Kits and Caspase-9 Fluorescence assay kits (both from Clontech). The above treated cells were harvested and lysed with cell lysis buffer for 10 min. After centrifuge, 50 μl of 2 Reaction Buffer/DTT Mix and 5 μl specific substrate of DEVD-AFC (for caspase-3) or LEHD-AMC (for caspase 9) were added to the supernatants for further incubation at 37°C. One hour later, caspase activities were detected by a fluorometer (model FL600, Bio-Tek Instruments).

### Assay of lactate dehydrogenase release

Lactate dehydrogenase (LDH) concentration was determined by a commercial kit (BioVision) according to the manufacturer's protocol. Briefly, cells were homogenized in ice-cold Assay buffer. Then, cells were centrifuged at 10000 × ***g*** for 15 min at 4°C. The collected supernatants were added to 50 μl Reaction Mix. About 30 min later, the absorbance was detected at 450 nm to evaluate LDL release.

### Analysis of mitochondrial membrane potential (Δ*ψ*_m_)

Cells were rinsed with PBS for three times and then re-suspended in fresh medium containing 1.0 μM of the mitochondrial probe rhodamine 123 reagent (Biodee Diagnostic), which is used to assess mitochondrial potential. After a 15 min incubation at 37°C in thermostatic bath, cells were washed and re-suspended in PBS buffer. Then, the samples were analysed by flow cytometry.

### Statistical analysis

All experiments were performed in triplicate. Data were analysed using SPSS 19.0 (SPSS). Results are shown as the mean ± S.D. All statistical analyses were evaluated using Student's *t* test and ANOVA. A value of *P*<0.05 was defined as statistically significant.

## RESULTS

### Down-regulation of Srx-1 in H9c2 cells following SI/R precondition

**Figure 1:**
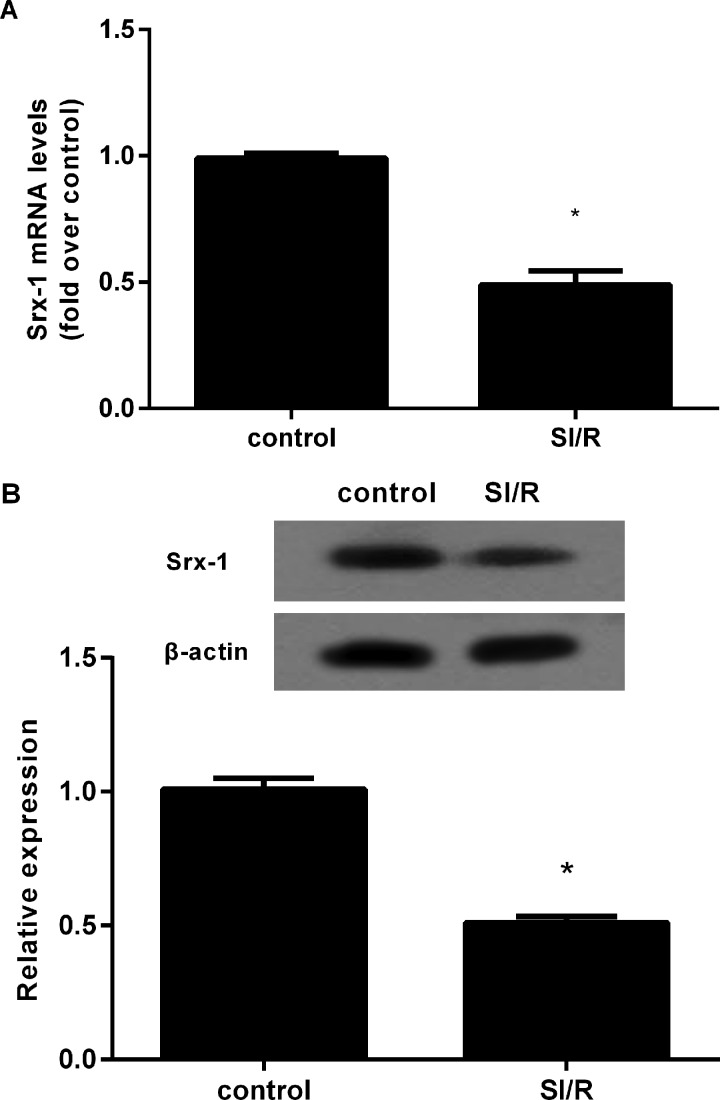
Down-regulation of Srx-1 in H9c2 cardiomyocytes under SI/R treatment The H9c2 cells were exposed to hypoxia for 10 h and then reoxygenated for 3 h. (**A**) The mRNA levels were detected by qRT-PCR. (**B**) The protein expression of Srx-1 was determined by western blotting. *P<0.05 compared with control groups.

To assess the potential effect of Srx-1 on myocardial ischaemia–reperfusion (I/R) injury, cells were treated with hypoxia–reoxygenation (H/R) to simulate I/R injury. RT-PCR analysis confirmed a dramatical decrease in Srx-1 mRNA levels under SI/R treatment, in contrast with control groups (Figure 1A). Consistently, further protein assay also demonstrated 0.49-fold down-regulation of Srx-1 expression after 10 h hypoxia and 3 h reoxygenation (SI/R), compared with control groups (Figure 1B). Accordingly, these results might suggest an aberrant decrease in Srx-1 under SI/R precondition, implying a potential important role in I/R injury.

### Overexpression of Srx-1 enhances cell viability under SI/R stimulation

**Figure 2:**
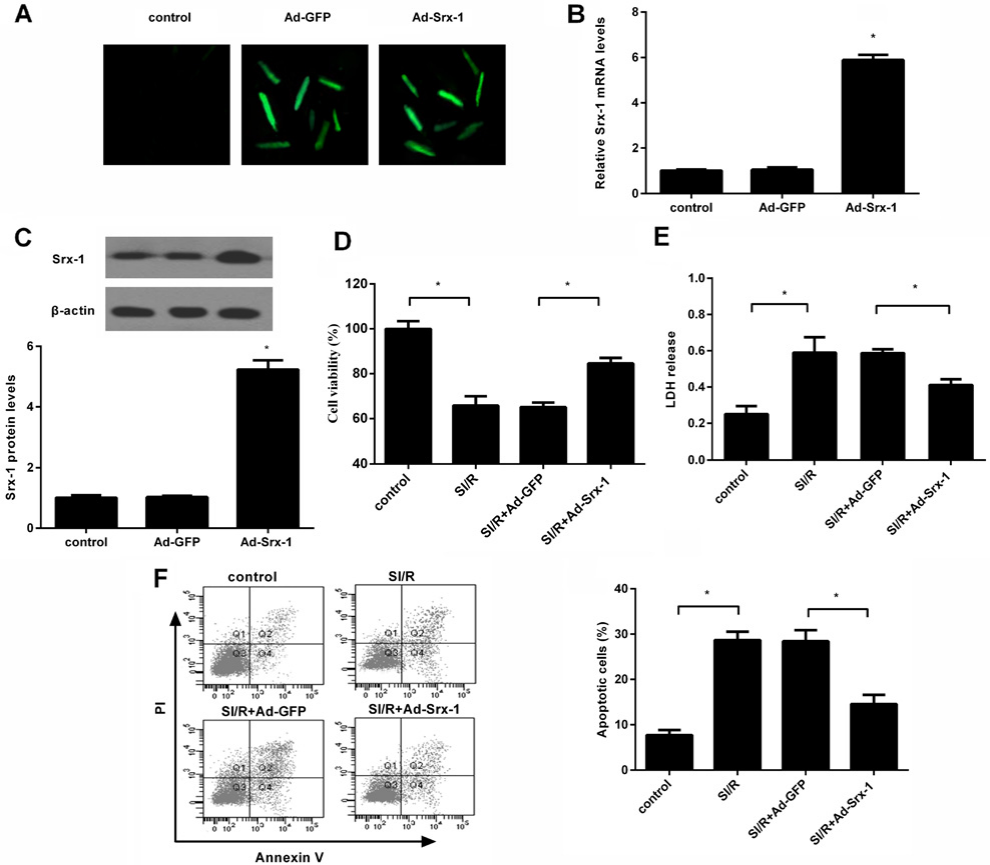
Effect of Srx-1 overexpression on SI/R-induced cell injury H9c2 cells were transfected with the 2 nmol/l of recombinant Ad-Srx-1 or Ad-GFP prior to subject to SI/R. (**A**) About 96% of cardiomyocytes were infected after 48 h adenoviral infection. (**B** and **C**) The effect on Srx-1 mRNA and protein levels were evaluated by qRT-PCR and western blotting. (**D**) About 0.5 mg/ml MTT solution was added to assess the effect on cell viability. (**E**) LDH concentration was determined using a colorimetric assay kit. (**F**) Cells were subsequently treated with Annexin V/PI staining. Typical representative of cell apoptosis under various treatments by flow cytometry assay. Graph showing cell apoptotic rate in H9c2 cells. *P<0.05.

Given the fact that Srx-1 was decreased under the condition of SI/R, the further function of Srx-1 on cell viability was evaluated. To investigate the above problem, cells were infected with the constructed recombinant Ad-Srx-1 or Ad-GFP. About 48 h later, we detected nearly 96% infection efficiency (Figure 2A). Further RT-PCR assay confirmed a significant increase in Srx-1 mRNA levels in H9c2 cells following Ad-Srx-1 infection (Figure 2B). Simultaneously, Ad-Srx-1 infection notably induced the expression of Srx-1 protein in H9c2 cells (Figure 2C). Function analysis corroborated that SI/R remarkably inhibited cell proliferation (Figure 2D). However, Srx-1 up-regulation strikingly ameliorated the inhibitory effect of SI/R precondition on cell proliferation from 65.78% to 84.21%, indicating a potential protective role of Srx-1 in cardiomyocytes under the condition of SI/R.

### Elevation of Srx-1 protects H9c2 cells from SI/R-induced cell apoptosis

To further explore the function of Srx-1 on SI/R-triggered cell injury, its effect on cell membrane damaging in H9c2 cells was detected. As shown in Figure 2(E), SI/R treatment significantly stimulated LDH leakage into the media, a biochemical index of cell death. Although overexpression of Srx-1 dramatically reduced the release of LDH triggered under SI/R condition, suggesting that Srx-1 could protect against SI/R-induced cell membrane injury. Further Annexin V-FITC and PI staining substantiated that SI/R dramatically triggered cell apoptosis and the apoptotic rate increased into 28.67% (Figure 2F), which was notably abated in Srx-1-overexpression groups. Therefore, these results confirmed that Srx-1 elevation might counteract the adverse effects of SI/R on cardiomyocyte growth.

### Silencing of Srx-1 aggravates cell injury upon SI/R

**Figure 3:**
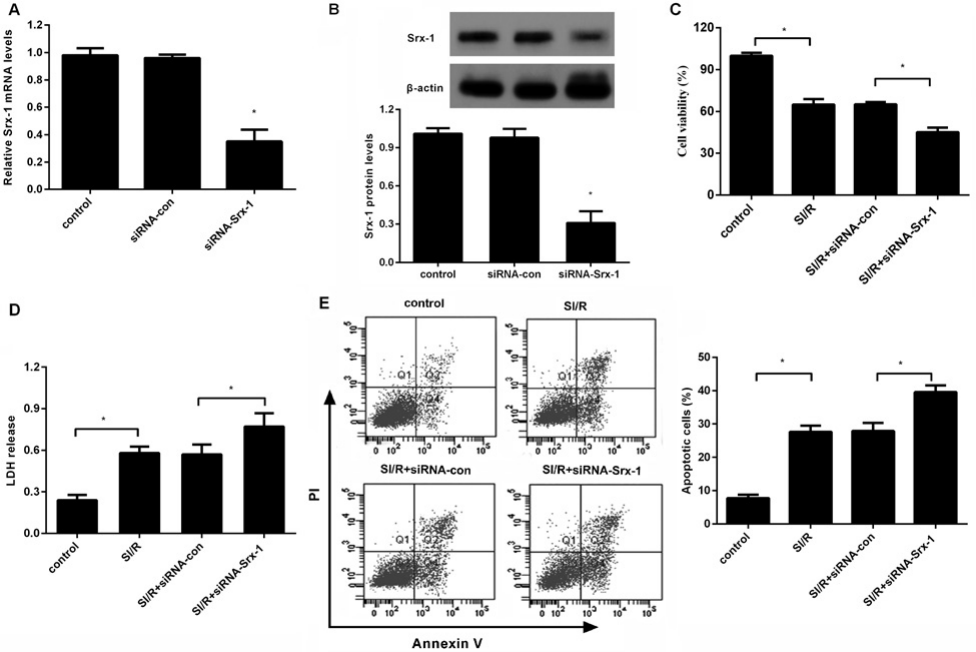
Silencing of Srx-1 accelerated cell injury upon SI/R (**A** and **B**) Following transfection with Srx-1 siRNA or siRNA-con, the mRNA and protein levels of Srx-1 were detected. (**C**) Effect of Srx-1 down-regulation on cell viability upon SI/R was evaluated by MTT assay. (**D**) Effect on LDH release. (**E**) Annexin V/PI staining was performed to determine cell apoptosis. *P<0.05.

To further investigate the effect of Srx-1 on SI/R-induced cell injury, we suppressed Srx-1 expression by Srx-1 siRNA transfection. As shown in Figure 3(A), Srx-1 siRNA treatment substantially reduced the mRNA levels of Srx-1, accompany with the similar decrease in Srx-1 protein expression (Figure 3B). Function assay demonstrated that knockdown of Srx-1 further augmented the restrained effect on cell viability of H9c2 cells under SI/R stimuli (Figure 3C). Moreover, SI/R-triggered release of LDH was potentiated when blocking Srx-1 expression (Figure 3D). Additionally, apoptotic rate induced by SI/R was further deteriorated after Srx-1 siRNA transfection (Figure 3E). These data suggested that Srx-1 silence aggravated cell injury under SI/R treatment, implying a potential protective function of Srx-1 on SI/R-induced cardiomyocyte injury.

### Srx-1 up-regulation attenuates mitochondrial apoptotic pathway in SI/R-treated H9C2 cells

**Figure 4:**
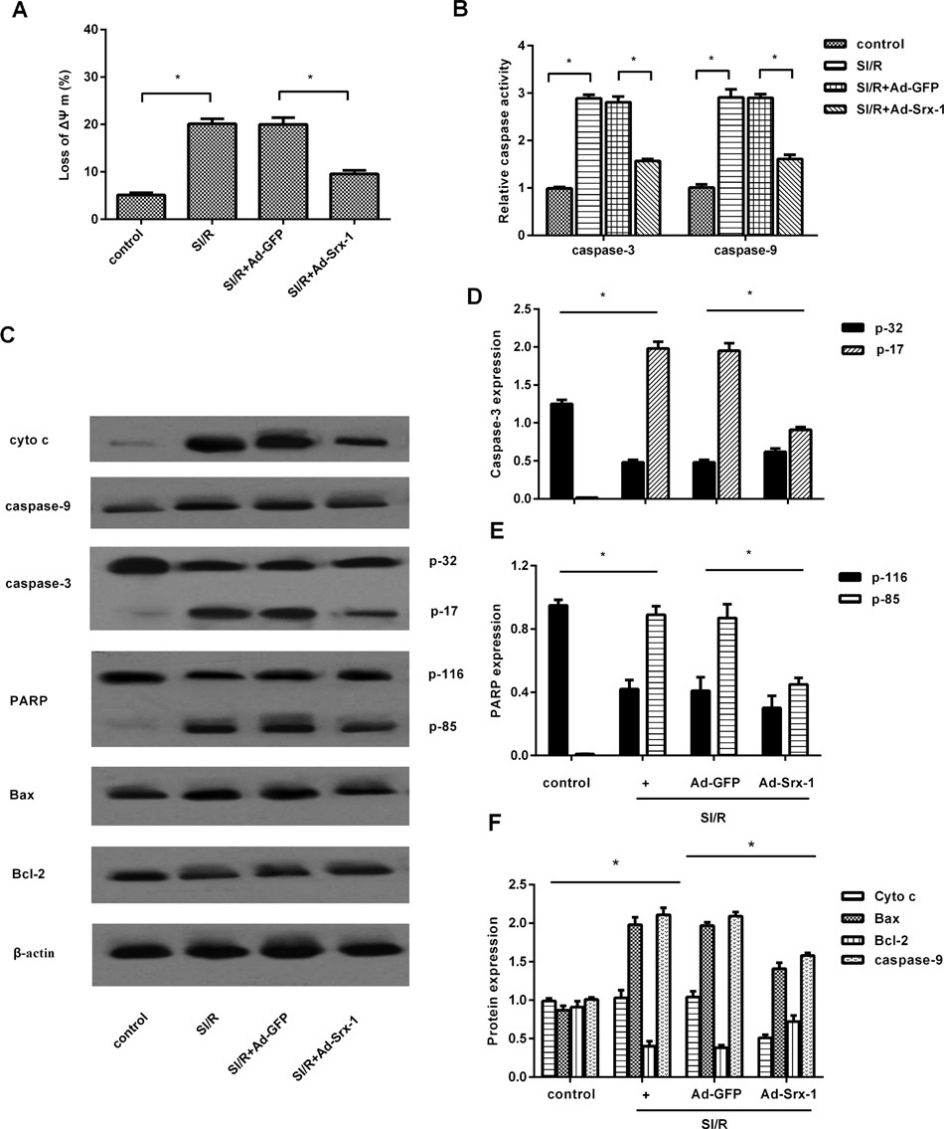
Elevation of Srx-1 attenuated SI/R-triggered mitochondrial apoptosis pathway After transfection with Ad-Srx-1, cells were then exposure to SI/R. (**A**) Following staining with rhodamine 123 reagent, the loss of Δ*ψ*_m_ was analysed by flow cytometry. (**B**) The specific substrates of DEVD-AFC (for caspase-3) or LEHD-AMC (for caspase-9) were introduced, and the caspase-9 and caspase-3 activity was assessed by fluorimetry. (**C**) Western blotting was performed to detect the expression of cytochrome *c*, caspase-9, caspase-3, PARP, Bax and Bcl-2. (**D**) Densitometry of western blot band intensities by Quantity One. *P<0.05.

It has been reviewed extensively that mitochondrial dysfunction contributes to the cell growth inhibitory response by triggering the events associated with the apoptotic pathway [18]. To clarify the mechanism underlying Srx-1-induced resistance to SI/R in H9c2 cells, the mitochondrial apoptotic signalling was explored. Following incubation under SI/R condition, the increase in the loss of Δ*ψ*_m_ was detected by Δ*ψ*_m_-sensitive rhodamine 123 (Figure 4A), indicating that mitochondria may participate in SI/R-triggered cell apoptosis. However, this decrease in Δ*ψ*_m_ was dramatically decreased when cells were infected with Ad-Srx-1. Simultaneously, SI/R preconditioning dramatically increased the activity (Figure 4B) and expression (Figure 4C) of caspase-9, an upstream initiator of caspase-3 signalling. Moreover, a similar increase in caspase-3 activity (Figure 4B) and expression (Figure 4C) was also validated upon SI/R. Nevertheless, these elevations were all mitigated following Ad-Srx-1 treatment. Importantly, the release of cytochrome *c* from mitochondria into the cytosol, an initial induction factor for cell apoptosis, was remarkably augmented under SI/R condition, which was dramatically decreased by Srx-1 overexpression (Figure 4C). Moreover, the subsequent increases in cleaved caspase-3 (p-17 kDa) (Figures 4C and 4D) and its substrate of PARP (p-85 kDa) expression were also reduced when Srx-1 was up-regulated in SI/R-treated cells. Furthermore, Srx-1 up-regulation also diminished the up-regulation of pro-apoptotic protein Bax expression in H9c2 cell upon SI/R treatment, concomitant with a down-regulation in the expression of anti-apoptotic protein Bcl-2 (Figures 4C and 4F). These results suggested that Srx-1 could protect cell from SI/R-induced cell death by regulating mitochondrial apoptotic pathway.

### PI3K/AKT signalling mediates the protective effect of Srx-1 on cell injury upon SI/R

**Figure 5:**
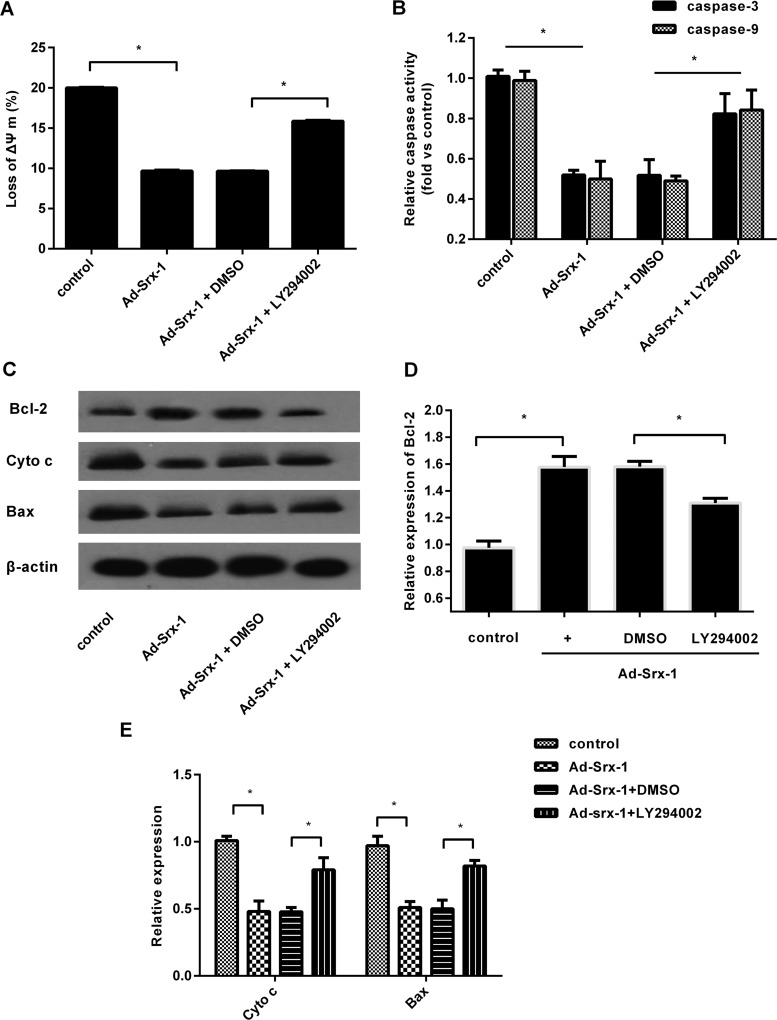
Srx-1 enhanced cardiomyocyte resistance to SI/R injury by the PI3K/AKT signalling (**A**) Western blotting analysis for phospho-AKT and AKT in H9c2 cell treated with Ad-Srx-1 upon SI/R. (**B**) Cells were precondition with the specific PI3K/AKT inhibitor LY294002 for 3 h, and then were transfected with Ad-Srx-1 under SI/R treatment. MTT assay were performed to determine cell viability. (**C**) Effect of LY294002 on Srx-1-inhibited LDH release. (**D**) The corresponding results in cell apoptosis by Annexin V-PI staining. *P<0.05.

Convincing evidences indicate that PI3K/AKT pathway exerts the prominent roles in multiple physiological processes of myocardial cells, including cell proliferation, apoptosis and migration [19,20]. To further elucidate the underlying mechanism involved in the protective role of Srx-1 in SI/R-treated cells, the activation of PI3K/AKT signalling was determined. As shown in Figure 5(A), SI/R stimulation significantly inhibited AKT phosphorylation (p-AKT), but not AKT expression. Although overexpression of Srx-1 significantly attenuated the above decrease in p-AKT expression, suggesting that Srx-1 might protect SI/R-mediated cell injury by activating the PI3K/AKT pathway. To further corroborate the correlation between PI3K/AKT and protective role of Srx-1 roles in SI/R-treated cells, MTT assay was performed. As expected, pretreatment with specific PI3K/AKT inhibitor LY294002 dramatically inhibited Srx-1-induced cell viability (Figure 5B). Concomitantly, the decrease in LDH concentration was dramatically attenuated (Figure 5C). Furthermore, LY294002 precondition also weakened the anti-apoptotic role of Srx-1 (Figure 5D). Therefore, these data confirmed that Srx-1 might exert its protective function in SI/R-induced cell injury by inducing the PI3K/AKT signalling.

### Srx-1 antagonizes SI/R-induced mitochondrial apoptotic pathway through the PI3K/AKT signalling

**Figure 6:**
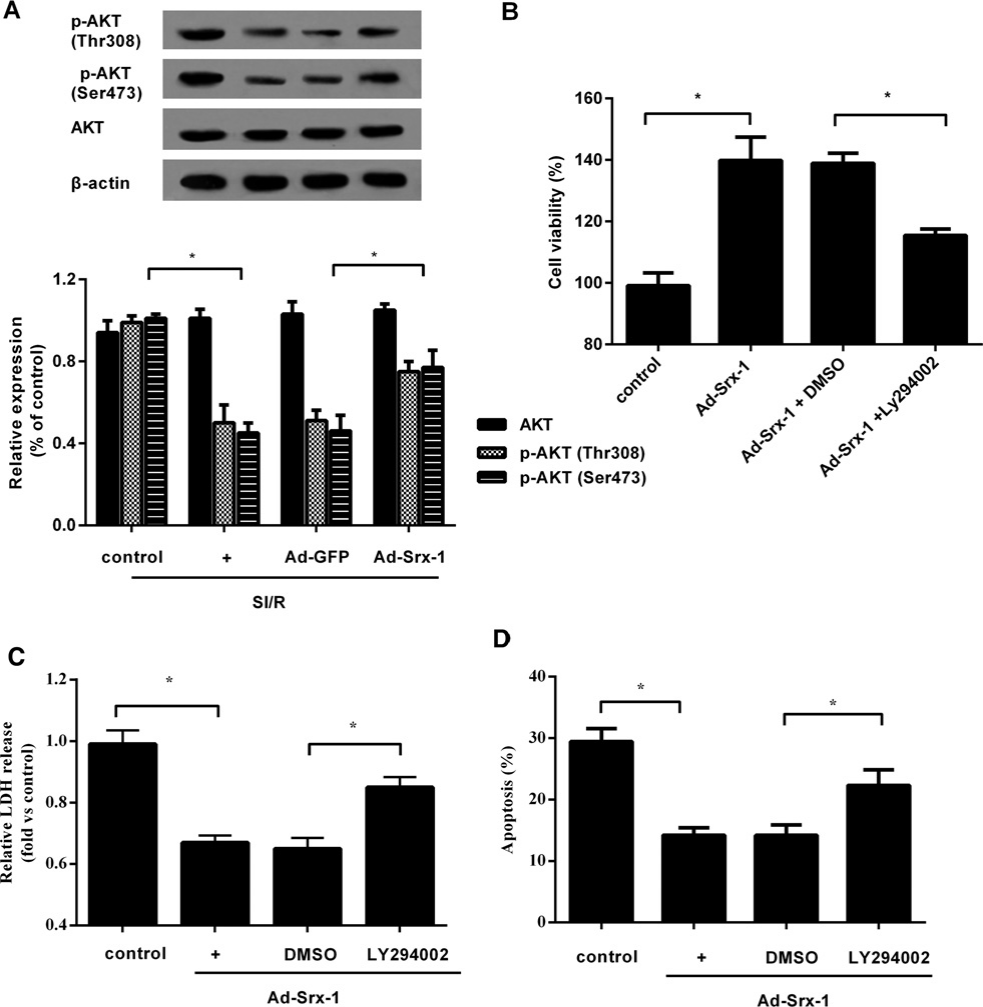
Involvement of the PI3K/AKT pathway in Srx-1-mediated cardiomyocyte resistance to mitochondrial apoptotic pathway triggered by SI/R condition Before transfection with Ad-Srx-1, H9c2 cells were pretreated with LY294002. Then, cells were incubated under 10 h hypoxia and 3 h reoxygenation (SI/R). (**A**) The effect on Δ*ψ*_m_ loss was analysed by flow cytometry. (**B**) The activity of caspase-9 and caspase-3 were evaluated by a fluorometer. (**C**) Western blotting assessed the expression of cytochrome *c*, Bax and Bcl-2. (**D**) The corresponding quantitative analysis of Bcl-2 protein. (**E**) The quantified band intensities of cytochrome *c* and Bax. *P<0.05.

Emerging studies have established that PI3K/AKT can negatively regulate the mitochondria intrinsic pathway [21,22]. To clarify the involvement between PI3K/AKT and Srx-1-mediated resistance to mitochondrial apoptosis pathway, cells were pre-treated with LY294002. The results showed that the inhibitory effect of Srx-1 on Δ*ψ*_m_ loss was dramatically decreased (Figure 6A). Moreover, the decrease in caspase-9 activity triggered by Srx-1 up-regulation was also mitigated following LY294002 precondition, accompany with the similar increase in subsequent caspase-3 activity (Figure 6B). Western blotting analysis conferred the down-regulation of cytochrome *c* in Srx-1 groups (Figure 6C). After pretreatment with LY294002, the decrease in cytochrome *c* expression was remarkably ameliorated. Simultaneously, Srx-1 elevation induced the 1.57-fold increase in Bcl-2 expression (Figures 6C and 6D), which was substantially reduced after LY294002 pretreatment. Consistently, LY294002 precondition also antagonized the inhibitory effect of Srx-1 on the expression of pro-apoptosis protein Bax (Figures 6C and 6E). Together, these experiments revealed that Srx-1 might enhance cell resistance to SI/R-induced mitochondrial apoptotic pathway through PI3K/AKT signalling.

## DISCUSSION

Ischaemia–reperfusion (I/R) injury within the myocardium is widely accepted as a major contributor for the development of ischaemic cardiovascular diseases, such as acute myocardial infarction and heart failure [8]. During this process, ROS accumulates in the post-ischaemic myocardium and this leads to severe cell injury, contributing to myocardial infarction [5]. Recently, accumulating studies have shown that restoring oxidative stress injury may be a potential strategy for the treatment of I/R-induced cardiovascular disease [12]. This research presents the prominent finding that the antioxidant Srx-1 was remarkably decreased in H9c2 cells upon SI/R. Importantly, its overexpression significantly ameliorated SI/R-triggered cell injury in a mitochondria apoptosis-dependent manner. Furthermore, PI3K/AKT signalling was confirmed to be involved in the above progression. Accordingly, this research may corroborate a protective role of Srx-1 up-regulation in SI/R-triggered cardiomyocyte injury by PI3K/AKT-mediated mitochondria apoptotic pathway.

Srx-1 is lately recognized as an endogenous antioxidant that protects against brain tissue damage in Parkinson's disease [23]. Furthermore, its silencing obviously decreases cell viability and its overexpression plays the anti-apoptotic and neuroprotective effects against oxidative stress-triggered cortical astrocyte injury, indicating a prominent role in cerebral ischaemia [13]. Nevertheless, its function in ischaemic cardiac diseases remains unclear. In the present study, we simulated I/R injury by exposing H9c2 cells to hypoxia for 10 h, followed by reoxygenation for 3 h. Of interest, an obvious down-regulation of Srx-1 was validated after cardiomyocyte I/R injury. To explore the potential effect of Srx-1 in SI/R-induced cardiomyocyte injury, Srx-1 elevation was modulated by Ad-Srx-1 transfection. Interestingly, Srx-1 overexpression dramatically attenuated the SI/R-induced cell cytotoxicity, accompany with the decrease in cell membrane damaging by detecting LDH levels. Simultaneously, SI/R-triggered cell apoptosis was remarkably decreased following Srx-1 up-regulation. Additionally, Srx-1 down-regulation dramatically deteriorated cell injury upon SI/R treatment. Therefore, the above data confirmed a protective role of Srx-1 in SI/R-induced cardiomyocyte injury, indicating a potential avenue against ischaemic heart diseases.

Given the fact that the oxidative stress-derived cell injury is associated with mitochondria-mediated apoptosis pathway [21]. During this progression, the cytochrome *c*/caspase-9 signalling is referred as the mitochondria-dependent intrinsic apoptosis pathway [18]. It is widely accepted that the mitochondrial membrane potential (Δ*ψ*_m_) plays a vital role in cell injury under conditions of ischaemia and hypoxia by regulating cytochrome *c* release [24,25]. In the present study, Srx-1 up-regulation inhibited the loss of Δ*ψ*_m_ and subsequent cytochrome *c* release from mitochondria into the cytosol triggered by SI/R stimulation. Importantly, Srx-1 reduced SI/R-induced caspase-9 activity and expression, an upstream regulator of caspase-3 signalling. Concomitantly, the activation of caspase-3 upon SI/R treatment was also noticeably mitigated following Srx-1 elevation, concomitant with the corresponding decrease in caspase-3 effector PARP activation, which eventually leading to cell apoptosis [26]. The pro-apoptotic and anti-apoptotic proteins of the Bcl-2 family is recognized as a pivotal regulator for mitochondria-dependent cell apoptotic pathway [27]. Here, SI/R treatment significantly up-regulated the expression of pro-apoptotic protein Bax and down-regulated the expression of anti-apoptotic protein Bcl-2, leading to Δ*ψ*_m_ collapse, cytochrome *c* release and subsequent activation of caspase-3 apoptosis pathway [28]. However, both of the above proteins were notably suppressed after Srx-1 overexpression. Together, these results show that Srx-1 may enhance the cell resistance to SI/R-induced cell injury by inhibiting the activation of mitochondria-mediated apoptotic pathway.

The PI3K/AKT signalling possesses the crucial roles in various physiological processes, such as cell proliferation, apoptosis and migration [19,21,22]. To further clarify the underlying mechanism of how Srx-1 mediates its protective effect on cardiomyocytes, we examined the role of PI3K/AKT pathway. In accordance with our hypothesis, Srx-1 overexpression enhanced the phosphorylation of AKT upon SI/R. When this pathway was blocked with LY294002, the pro-proliferation effect of Srx-1 was attenuated. Simultaneously, LY294002 precondition also antagonized the anti-apoptosis role of Srx-1. These results suggested that Srx-1 might protect cardiomyocyte from SI/R injury by regulating the PI3K/AKT pathway. Recently, PI3K/AKT signalling was found to be reversely associated with mitochondria dependent apoptosis [21,22]. Presently, blocking the PI3K/AKT pathway with LY294002 attenuated the inhibitory effect of Srx-1 on Δ*ψ*_m_ loss. Furthermore, Srx-1-induced decreases in cytochrome *c* release, caspase-9 and caspase-3 activity and Bax expression were significantly abated after LY294002 precondition, accompany with the down-regulation of Bcl-2 levels. Together, the above data corroborated that Srx-1 might elicit the protective role in I/R-induced cardiomyocyte injury by regulating the PI3K/AKT-mediated mitochondria apoptotic pathway.

In conclusion, the present study demonstrated the down-regulation of Srx-1 under SI/R condition. Furthermore, overexpression of Srx-1 ameliorated SI/R-induced cell injury by blocking mitochondria apoptotic pathway. Moreover, the protective role of Srx-1 in SI/R-triggered mitochondria apoptosis was mediated through the PI3K/AKT signalling. Accordingly, this research may support a promising therapeutic avenue for the treatment of ischaemic cardiovascular diseases. The mechanistic investigations *in vivo* will be explored in future studies.

## Abbreviations

GFP: green fluorescent protein
LDH: lactate dehydrogenase
PARP: poly (ADP ribose) polymerase
ROS: reactive oxygen species
SI/R: simulated ischaemia–reperfusion
Srx-1: sulfiredoxin-1
Δ*ψ*_m_: mitochondrial membrane potential
